# Normotensive and hypertensive Immunoglobulin a nephropathy with ischemic renal injury: clinicopathological characteristics and prognosis

**DOI:** 10.1080/0886022X.2021.1994996

**Published:** 2021-10-27

**Authors:** Yongli Wang, Xutong Wang, Dan Yu, Minhua Xie, Jingjing Ren, Yuze Zhu, Haonan Guo, Songxia Quan, Junjun Zhang

**Affiliations:** a Department of Nephrology, The First Affiliated Hospital of Zhengzhou University, Zhengzhou, People’s Republic of China; b Department of Renal Pathology, The First Affiliated Hospital of Zhengzhou University, Zhengzhou, People’s Republic of China

**Keywords:** Blood pressure, IgA nephropathy, ischemic renal injury, prognosis, renal biopsy

## Abstract

**Objectives:**

This study aimed to investigate the clinicopathological characteristics and prognosis of normotensive and hypertensive IgAN patients with ischemic renal injury.

**Methods:**

A total of 344 cases of IgAN with ischemic renal injury were included in the study, including 99 normotensive IgAN patients (28.8%) and 245 hypertensive IgAN patients (71.2%). In addition, 467 IgAN patients without ischemic renal injury were included as controls, including 205 normotensive patients and 262 hypertensive patients. Clinicopathological and prognostic data were collected and analyzed.

**Results:**

Compared with patients without ischemic renal injury, IgAN patients with ischemic renal injury displayed a higher proportion of hypertention, a higher proportion of ischemic glomerulosclerosis, tubular atrophy/interstitial fibrosis and vascular lesions (all *p* < .05). There was no significant difference in cumulative survival between the normotensive IgAN patients groups (Log-rank χ^2^ = 0.479; *p* = .489). Furthermore, ischemic renal injury was not a risk factor for end-point events in normotensive IgAN patients (HR = 1.103; 95% CI: 0.279–4.365; *p* = .889). There was lower cumulative survival in hypertensive IgAN patients with ischemic renal injury (Log-rank χ^2^ = 11.352, *p* = .001). Moreover, ischemic renal injury was a risk factor for end-point events in hypertensive IgAN patients (HR = 1.889; 95% CI: 1.124–3.178; *p* = .016).

**Conclusions:**

Ischemic renal injury can occur in normotensive IgAN patients. Although the pathological changes may not affect the long-term prognosis of normotensive IgAN patients, the prognosis for hypertensive IgAN patients remains poor. Therefore, increased attention should be paid to the clinical management of ischemic lesions in hypertensive IgAN patients.

Immunoglobulin A nephropathy (IgAN), a form of mesangial proliferative glomerulonephritis, is characterized by the predominant deposition of IgA in the glomerular mesangium. The most common primary glomerulonephritis globally is IgAN. IgAN also represents among the most frequent causes of primary glomerulonephritis in China, constituting approximately 45% of all primary glomerulonephritis cases and remaining the primary cause of end-stage kidney disease (ESKD) [1]. Pathologically, a spectrum of glomerular manifestations has been recorded. However, mesangial proliferation with IgA or IgA-based immune complex deposition in the mesangial region is prominently evident [2]. Within 20 years of the onset of the disease, almost 36% of adult patients with IgAN may progress to ESKD in China [3]. Ischemic renal injury is characterized by arteriolar wall thickening, hyaline degeneration, and luminal stenosis, and is accompanied by ischemic glomerular sclerosis and renal tubular atrophy/interstitial fibrosis [4]. Ischemic renal injury is more frequent in IgAN patients with hypertension, and accumulating evidence suggests that it is closely associated with hypertension [5]. However, a number IgAN patients with normal blood pressure may also have ischemic renal injury. The influence of ischemic renal injury on the prognosis of IgAN patients without hypertension has rarely been investigated. In the present study, we retrospectively analyzed the clinicopathologic characteristics and prognosis of IgAN patients with ischemic renal injury, and using subgroup analysis for hypertensive and normotensive patients with or without hypertension, to raise awareness of the disease.

## Materials and methods

### Statement of ethics

The present study was conducted in accordance with the Declaration of Helsinki and was approved by the Institutional Review Board of The First Affiliated Hospital of Zhengzhou University (approval number: 2020-KY-475), Zhengzhou, China. The requirement for informed consent from the participants was waived due to the retrospective nature of the study.

### Patient selection

A total of 11647 patients undergoing a renal biopsy at the Department of Nephrology of The First Affiliated Hospital of Zhengzhou University between 1 January 2015, and 30 December 2017, including 3458 patients were diagnosed with IgA nephropathy.

Inclusion criteria were as follows: patients with histopathologically-confirmed primary IgAN with or without ischemic kidney injury (Diagnostic criteria for ischemic renal injury: Under a light microscope, the glomeruli are observed to have ischemic shrinkage, ischemic sclerosis, with or without tubular atrophy/interstitial fibrosis. The number of ischemic sclerotic glomeruli should account for more than 25% of the total.); age at the time of biopsy ≥18 years; follow-up exceeding 12 months; estimated GFR (eGFR) ≥15 mL/min/1.73 m^2^; and a biopsy specimen with a minimum of 10 total glomeruli. However, patients with secondary causes of mesangial IgA deposits, including Henoch–Schönlein purpura, nephritis, or hepatitis B or C, or those with comorbidities, such as diabetes mellitus, membranous nephropathy, or Alport syndrome were excluded from the study. According to the entry and exclusion criteria, 811 eligible IgAN patients were analyzed in this retrospective cohort study, including 344 cases of IgAN with ischemic renal injury. Of the 344 patients, 99 (28.8%) had normal blood pressure, and 245 patients (71.2%) were hypertensive. In addition, we also included 467 cases of IgAN without ischemic renal injury as controls, including 205 with normal blood pressure and 262 with hypertension.

### Data collection

The following clinical data were collected at the time of renal biopsy: age, gender, BMI and blood pressure. Routine laboratory investigation data were obtained, including blood biochemistry, 24-h urine protein, and microscopic hematuria. Follow-up data was collected by outpatient review, which comprised eGFR (using the Chronic Kidney Disease Epidemiology Collaboration equation; CKD-EPI) [6]. According to 2012 KDIGO guidelines [7], angiotensin-converting enzyme inhibitors (ACEI) or angiotensin receptor blockers (ARB) can be used as a treatment for proteinuria and high blood pressure, and so can also be used as a treatment for proteinuria in IgAN patients with normal blood pressure. In addition, glucocorticoid therapy is recommended for IgAN patients with eGFR >50 mL/min/1.73 m^2^ after 3–6 months of optimal supportive treatment with albuminuria ≥1 g/d. Immunosuppressive therapy was used in the case of insensitivity and a contraindication against glucocorticoid therapy. The follow-up data also comprised the medication history for ACEI/ARB, corticosteroids, and immunosuppressive agent intervention (tacrolimus, mycophenolate mofetil, *Tripterygium wilfordii glycosides*). All renal biopsies were processed for immunofluorescence and light and electron microscopy using routine techniques, as described previously, and evaluated by two experienced renal pathologists. Renal pathology was scored according to the updated Oxford Classification for IgAN [8]. MESTC scores were assessed as follows: mesangial hypercellularity (M0: mesangial score ≤0.5; M1: mesangial score >0.5), endocapillary hypercellularity (E0: absent; E1: present), segmental glomerulosclerosis (S0: absent; S1: present), tubular atrophy/interstitial fibrosis (T0: ≤25%; T1: 26%–50%; T2: >50%), and cellular and fibrocellular crescentic lesions (C0: =0; C1: >0 and <25%; C2: ≥25%). Vascular lesions was also scored in this study (0: no lesions; 1: arteriolar wall thickening/lumen narrowing; 2: merging glassy changes).

### Definitions

Definition of the hypertension: Hypertension was defined in accordance with the 2018 Chinese guidelines for the prevention and treatment of hypertension, namely as a previous clear diagnosis, or a clinical systolic pressure ≥140 mmHg or diastolic pressure ≥90 mmHg measured 3 times when in a resting state during hospitalization [9].

Definition of the renal outcome: Follow-up time was defined as the duration from pathological diagnosis to final follow-up; End-points were defined as a permanent 50% reduction in eGFR compared with the baseline value, or progression to ESKD.

### Statistical analyses

All statistical analyses were conducted using SPSS software (version 21.0). Continuous variables that were normally distributed were expressed as means ± standard deviation (SD), while those not normally distributed were expressed as medians and interquartile ranges (25%, 75%). Student’s *t*-tests and chi-squared tests were used to compare normally distributed continuous data and disordered categorical variable data. Mann–Whitney *U*-tests were used for non-normally distributed continuous data and for ordered categorical variable data. Log-rank tests were used to compare poor renal event-free survival. Cox regression analyses were performed to analyze the clinical predictors of poor prognosis. All tests were two-tailed. *p*-values < .05 were considered statistically significant.

## Results

### Representative images of H&E, PAS, and Masson’s staining for IgAN patients with ischemic renal injury are presented in Figure 1

**Figure 1.**
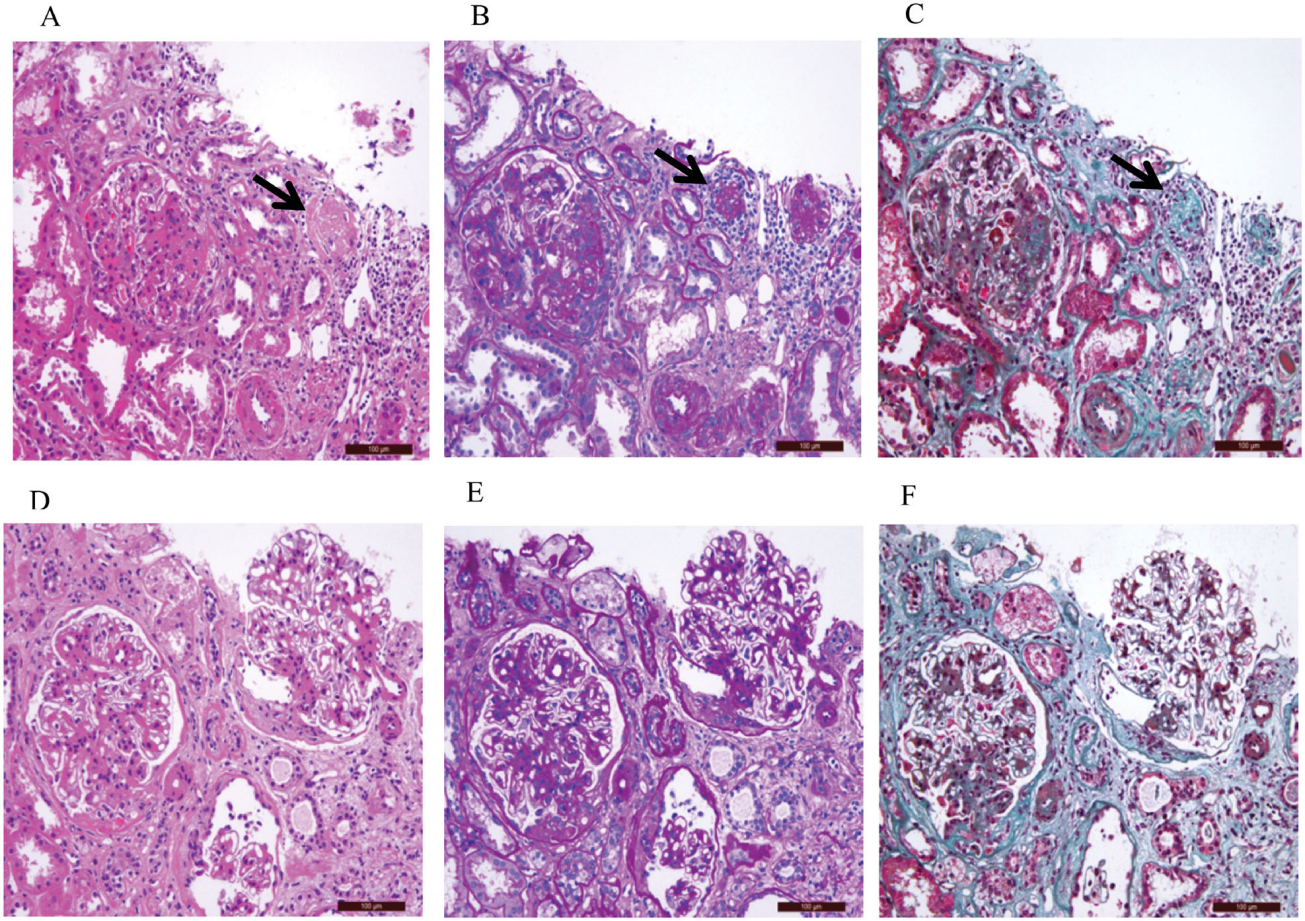
Representative images of the renal biopsy histopathology of IgAN patients with ischemic renal injury (A–C), IgAN patients without ischemic renal injury (D - F); H&E staining (200x). (A, D); PAS staining (200x). (b, e); Masson staining(200x). (C, F).

The renal histopathology of IgAN patients with ischemic renal injury indicated the presence of ischemic glomerulosclerosis (Black arrow) on images of H&E (Figure 1(A)), PAS (Figure 1(B)), and Masson’s staining (Figure 1(C)). In addition, renal interstitial inflammatory cell infiltration, tubule atrophy, and interstitial fibrosis can be observed in samples from IgAN patients with ischemic renal injury. The renal histopathology of IgAN patients without ischemic renal injury demonstrated no ischemic glomerulosclerosis. In addition, IgAN patients without ischemic renal injury also demonstrated the presence of renal interstitial inflammatory cell infiltration, tubule atrophy, and interstitial fibrosis (Figure 1(D–F)).

### Clinicopathological characteristics and prognosis of all IgAN patients with ischemic renal injury

A total of 811 IgAN patients were evaluated, of which 344 (42.4%) suffered ischemic renal injury. Compared with patients without ischemic renal injury, those with ischemic renal injury displayed a higher proportion of hypertension, higher levels of creatinine and uric acid (*p* < .05). Renal pathology revealed a higher proportion of ischemic glomerulosclerosis, a higher proportion of tubular atrophy/interstitial fibrosis (T1/T2 lesions) and vascular lesions(1/2) (all *p* < .05) in patients with ischemic renal injury (Table 1).

**Table 1.** Characteristics of the patients (*N* = 811).

Characteristics	With ischemic renal injury (*n* = 344)	Without ischemic renal injury (*n* = 467)	*p* value
Men	177 / 344	261 / 467	.211
Age, years	35 (29, 44)	34 (27, 44)	.053
Systolic pressure, mmHg	135 (125, 146)	131 (121, 141)	.001
Diastolic pressure, mmHg	89 (80, 98)	86 (79, 95)	.001
Hypertension, *n*(%)	245 (71.2)	262 (56.1)	<.001
BMI, kg/m^2^	26.53 (24.15, 28.74)	26.53 (24.15, 28.61)	.750
Hemoglobin, g/L	130.71 ± 19.53	131.36 ± 18.04	.621
Blood glucose, mmol/L	4.63 (4.23, 5.06)	4.59 (4.22, 5.03)	.793
Serum creatinine, umol/L	95.0 (75.0, 146.0)	77.5 (63.0, 102.0)	<.001
Serum uric acid, umol/L	372.51 ± 107.99	335.85 ± 102.27	<.001
Urea nitrogen, mmol/L	6.10 (4.66, 8.70)	5.30 (4.30, 6.90)	<.001
Total cholesterol, mmol/L	4.79 (4.09, 5.59)	4.80 (4.04, 5.69)	.975
Triglycerides, mmol/L	1.53 (1.10, 2.33)	1.36 (0.97, 2.16)	.004
EGFR	77.98 (49.98, 101.92)	97.21 (72.97, 115.28)	<.001
Serum albumin, g/L	39.75 (36.20, 43.40)	40.10 (36.10, 43.10)	.944
Urine protein, g/24h	1.76 (1.00, 2.74)	1.64 (0.85, 2.45)	.095
Urine erythrocyte, /mL	40.1 (12.5, 117.0)	44.0 (11.0, 144.0)	.410
Ischemic glomerulosclerosis, %	33.33 (23.08, 45.23)	3.77 (0.00, 9.70)	<.001
M1	88 (25.6)	80 (17.1)	.136
E1	114 (33.1)	77 (16.5)	.558
S1	236 (68.6)	290 (62.1)	.063
T1/T2	74 (21.5) / 83 (24.1)	60 (12.8) / 45 (9.6)	<.001
C1/C2	115 (33.4) / 6 (1.7)	142 (30.4) / 7 (1.5)	.366
Vascular lesions, 1/2	121 (35.2) / 138 (38.4)	132 (29.6) / 141 (30.2)	<.001
ACEI/ARB, *n*(%)	258 (75.0)	352 (75.4)	.934
Glucocorticoids/immunosuppressors, *n*(%)	181 (52.6)	224 (48.0)	.201
Follow-up duration, months	25.0 (17.0, 32.0)	26.0 (20.0, 34.0)	.122
ESKD, *n*(%)	44 (12.8)	27 (5.8)	–
Endpoint event, *n*(%)	55 (16.0)	33 (7.1)	–

BMI: Body Mass Index; EGFR: estimated glomerular filtration rate; Units: 15 mL/min/1.73 m^2^; M: mesangial hypercellularity; E: endocapillary hypercellularity; S: segmental glomerulosclerosis; T: tubular atrophy/interstitial fifibrosis; C: cell/cell fiber crescentic lesions; Vascular lesions 1: arteriolar wall thickening/lumen narrowing; 2: merge glassy changes; ACEI/ARB: Angiotensin-Converting Enzyme Inhibitors/Angiotensin Receptor Blockers; ESKD: end-stage kidney disease; Endpoint event: a 50% decrease in eGFR or ESKD; The categorical variable data was expressed as a rate (%). Student’s *t*-test or the chi-square test was used to compare the values of the two groups.

**Figure 2.**
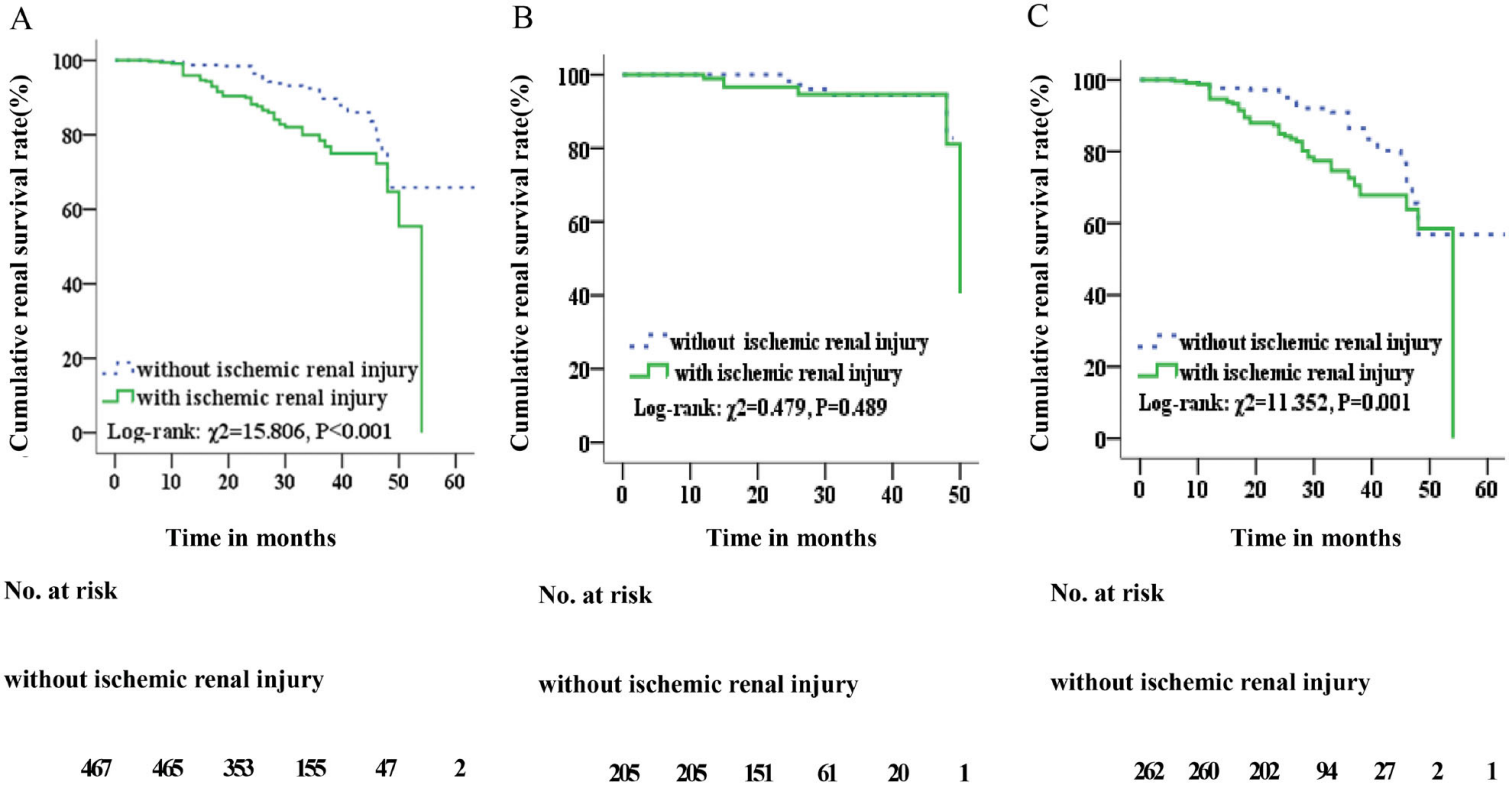
Kaplan-Meier analysis. (A) Comparison of renal cumulative survival with and without ischemic renal injury in IgA nephropathy patients. (B) Comparison of renal cumulative survival with and without ischemic renal injury in normotensive IgA nephropathy patients. (C) Comparison of renal cumulative survival with and without ischemic renal injury in hypertensive IgA nephropathy patients.

The median follow-up period was 25.0 months for patients with ischemic renal injury. End-point events occurred in 55 (16.0%) patients with ischemic renal injury, comprising 44 cases (12.8%) with ESKD. In comparison, the median follow-up was 26.0 months for patients without ischemic renal injury. The end-point event occurred in 33 cases (7.1%) without ischemic renal injury, comprising 27 cases (5.8%) with ESKD (Table 1). Kaplan-Meier survival analysis revealed that patients with ischemic renal injury exhibited lower cumulative renal survival (Log-rank χ^2^ = 15.806; *p* < .001) (Figure 2(A)). Adjusting for gender, age, proteinuria, renal function, MESTC pathology, and treatment, ischemic renal injury was found to be a risk factor for renal prognosis in IgAN patients (HR = 1.906; 95% CI: 1.189–3.054; *p* = .007) (Table 2).

**Table 2.** The association between ischemic renal injury and poor prognosis in all IgA nephropathy patients.

Model	HR	95%CI	*p* value
Model 1	2.387	1.547–3.681	<.001
Model 2	1.672	1.082–2.585	.021
Model 3	1.925	1.205–3.075	.006
Model 4	1.906	1.189–3.054	.007

Model 1: Univariate analysis showed that the age and sex of IgA nephropathy patients with ischemic renal injury and non-ischemic renal injury were matched; Model 2: eGFR and 24 h urinary protein were corrected on the basis of model 1; Model 3: mesangial and endocapillary hypercellularity, segmental glomerulosclerosis, and tubular atrophy/interstitial fifibrosis, and cell/cellular fibrous crescents were corrected on the basis of model 2; Model 4: the treatment of Angiotensin-Converting Enzyme Inhibitors/Angiotensin Receptor Blockers or glucocorticoids/immunosuppressors were corrected on the basis of model 3.

### Clinicopathological characteristics and prognosis of normotensive IgAN patients with ischemic renal injury

A total of 304 normotensive IgAN patients were evaluated, of which 99 (32.6%) suffered ischemic renal injury. Compared with patients without ischemic renal injury, those with ischemic renal injury displayed a lower eGFR, and higher levels of creatinine and uric acid (*p* < .05). However, there were no significant differences in serum albumin and 24 h urine protein (*p* > .05). Renal pathology revealed a higher proportion of ischemic glomerulosclerosis (31.81% (22.72%, 44.73%) vs. 4.17% (0, 9.52%); *p* < .05), a higher proportion of tubular atrophy/interstitial fibrosis (T1/T2 lesions) (14 (14.1%)/10 (10.1%) cases vs. 17 (8.3%)/9 (4.4%) cases; *p* < .05) and vascular lesions(1/2) (31 (31.3%)/31 (31.3%) cases vs. 54 (26.3%)/42 (20.5%) cases; *p* < .05) in patients with ischemic renal injury (Table 3). It should be noted that although there was a statistical difference in ischemic glomerulosclerosis and T lesions between the two groups, the proportion, and severity of ischemic glomerulosclerosis and T lesions in normotensive patients with ischemic renal injury was lower than those in hypertensive patients with ischemic renal injury.

**Table 3.** Characteristics of the patients with normal blood pressure (*N* = 304).

Characteristics	With ischemic renal injury (*n* = 99)	Without ischemic renal injury (*n* = 205)	*p* value
Men	42 / 99	100 / 205	.328
Age, years	35.0 (27.0, 43.5)	30.0 (25.0, 40.0)	.013
Systolic pressure, mmHg	125 (115, 130)	122 (113, 129)	.168
Diastolic pressure, mmHg	80 (76, 85)	79 (74, 85)	.242
BMI, kg/m^2^	27.15(24.35, 29.78)	27.03(24.34, 28.74)	.498
Hemoglobin, g/L	127.0 (119.5, 139.5)	127.0 (120.0, 140.5)	.562
Blood glucose, mmol/L	4.56 ± 0.68	4.58 ± 0.62	.819
Serum creatinine, umol/L	76.0 (58.5, 98.5)	70.0 (59.0, 85.5)	.036
Serum uric acid, umol/L	335.82 ± 110.37	300.18 ± 91.49	.003
Urea nitrogen, mmol/L	5.10 (4.20, 6.15)	4.90 (3.70, 5.90)	.063
Total cholesterol, mmol/L	4.83 (4.27, 5.70)	4.81 (4.02, 5.71)	.427
Triglycerides, mmol/L	1.23 (0.97, 2.00)	1.09 (0.82, 1.96)	.086
EGFR	92.27 (78.53, 115.73)	108.90 (89.27, 119.26)	.004
Serum albumin, g/L	39.40 (36.15, 43.00)	39.85 (35.55, 42.55)	.880
Urine protein, g/24h	1.64 (0.80, 2.14)	1.49 (0.80, 2.12)	.946
Urine erythrocyte, /mL	47.52 (15.50, 132.48)	78.50 (15.18, 240.00)	.087
Ischemic glomerulosclerosis, %	31.81 (22.72, 44.73)	4.17 (0.00, 9.52)	<.001
M1	24 (24.2%)	37 (18.1%)	.223
E1	31 (31.3%)	63 (30.7%)	1.000
S1	66 (66.7%)	124 (60.5%)	.314
T1/T2	14 (14.1%) / 10 (10.1%)	17 (8.3%) / 9 (4.4%)	.010
C1/C2	40 (40.4%) / 2 (2.0%)	78 (38.1%) / 1(0.5%)	.459
Vascular lesions, 1/2	31(31.3%) / 31 (31.3%)	54 (26.3%) / 42 (20.5%)	.010
ACEI/ARB, *n*(%)	73 (73.7)	143 (69.8)	.503
Glucocorticoids/immunosuppressors, *n*(%)	47 (47.5)	99(48.3)	1.000
Follow-up duration, months	25.0 (16.0, 32.0)	25.0(18.5, 32.0)	.827
ESKD, *n*(%)	5 (5.1)	5(2.4)	–
Endpoint event, *n*(%)	6 (6.1)	6(2.9)	–

BMI: Body Mass Index ; EGFR: estimated glomerular filtration rate; Units: 15 mL/min/1.73 m^2^; M: mesangial hypercellularity; E: endocapillary hypercellularity; S: segmental glomerulosclerosis; T: tubular atrophy/interstitial fifibrosis; C: cell/cell fiber crescentic lesions; Vascular lesions 1: arteriolar wall thickening/lumen narrowing; 2: merge glassy changes; ACEI/ARB: Angiotensin-Converting Enzyme Inhibitors/Angiotensin Receptor Blockers; ESKD: end-stage kidney disease; Endpoint event: a 50% decrease in eGFR or ESKD; The categorical variable data was expressed as a rate (%). Student’s *t*-test or the chi-square test was used to compare the values of the two groups.

The median follow-up period was 25.0 months for both groups. End-point events occurred in 6 (6.1%) patients with ischemic renal injury, comprising 5 cases (5.1%) with ESKD. The end-point event occurred in 6 cases (2.9%) without ischemic renal injury, comprising 5 cases (2.4%) with ESKD (Table 3). Kaplan-Meier survival analysis revealed that there was no significant difference in cumulative renal survival rates between the two groups (Log-rank χ^2^ = 0.479; *p* = .489) (Figure 2(B)). Adjusting for gender, age, proteinuria, renal function, MESTC pathology, and treatment, ischemic renal injury was not found to be a risk factor for renal prognosis in normotensive IgAN patients (HR = 1.103; 95% CI: 0.279–4.365; *p* = .889) (Table 4).

**Table 4.** The association between ischemic renal injury and poor prognosis in IgA nephropathy patients with normal blood pressure.

Model	HR	95%CI	*p* value
Model 1	1.438	0.434–4.764	.552
Model 2	0.978	0.265–3.611	.974
Model 3	1.280	0.308–5.320	.734
Model 4	1.103	0.279–4.365	.889

Model 1: Univariate analysis showed that the age and sex of IgA nephropathy patients with ischemic renal injury and non-ischemic renal injury were matched; Model 2: eGFR and 24 h urinary protein were corrected on the basis of model 1; Model 3: mesangial and endocapillary hypercellularity, segmental glomerulosclerosis, and tubular atrophy/interstitial fifibrosis, and cell/cellular fibrous crescents were corrected on the basis of model 2; Model 4: the treatment of Angiotensin-Converting Enzyme Inhibitors/Angiotensin Receptor Blockers or glucocorticoids/immunosuppressors were corrected on the basis of model 3.

### Clinicopathologic characteristics and prognosis of hypertensive IgAN patients with ischemic renal injury

A total of 507 hypertensive IgAN patients were evaluated, including 245 cases (48.3%) with ischemic renal injury. Compared with patients without ischemic renal injury, those with ischemic renal injury displayed a lower estimated eGFR, higher levels of creatinine, and uric acid (*p* < .05). Renal pathology suggested that a higher proportion of ischemic glomerulosclerosis (34.61% (25.59%, 50.00%) vs. 3.22% (0, 10.00%); *p* < .001), a higher proportion of T1/T2 lesions (60 (24.5%)/73 (29.8%) cases vs. 43 (16.4%)/36 (13.7%) cases; *p* < .05) and vascular lesions(1/2) (90 (36.7%)/101 (41.2%) cases vs. 84 (32.1%)/99 (37.8%) cases; *p* < .05) in patients with ischemic renal injury (Table 5).

**Table 5.** Characteristics of the patients with hypertension (*N* = 507).

Characteristics	With ischemic renal injury (*n* = 245)	Without ischemic renal injury (*n* = 262)	*p* value
Men	135 / 245	161 / 262	.151
Age, years	36.0 (30.0, 45.0)	37.5 (29.0, 46.0)	.651
Systolic pressure, mmHg	140 (131, 152)	140 (131, 153)	.929
Diastolic pressure, mmHg	93 (88, 100)	94 (87, 100)	.934
BMI, kg/m^2^	26.37(24.13, 28.61)	26.34(24.02, 28.54)	.686
Hemoglobin, g/L	131.86 ± 20.84	133.51 ± 18.29	.345
Blood glucose, mmol/L	4.68 (4.23, 5.12)	4.66 (4.27, 5.12)	.731
Serum creatinine, umol/L	113.0 (82.0, 159.5)	88.0 (70.0, 118.0)	<.001
Serum uric acid, umol/L	387.33 ± 103.61	363.62 ± 101.75	.010
Urea nitrogen, mmol/L	6.75 (5.06, 9.40)	5.83 (4.61, 7.52)	<.001
Total cholesterol, mmol/L	4.77 (4.04, 5.54)	4.79 (4.06, 5.69)	.648
Triglycerides, mmol/L	1.75 (1.13, 2.45)	1.62 (1.13, 2.27)	.233
EGFR	70.07 (40.91, 95.69)	88.69 (57.87, 107.99)	<.001
Serum albumin, g/L	40.1 (36.2, 43.5)	40.1 (36.3, 43.5)	.793
Urine protein, g/24h	1.95 (1.10, 3.00)	1.71 (0.93, 2.70)	.150
Urine erythrocyte, /mL	37.00 (11.00, 113.50)	29.85 (10.00, 100.98)	.268
Ischemic glomerulosclerosis, %	34.61 (25.59, 50.00)	3.22 (0.00, 10.00)	<.001
M1	56 (22.9)	51 (19.5)	.384
E1	46 (18.8)	51 (19.5)	.910
S1	170 (69.4)	166 (63.4)	.159
T1/T2	60 (24.5) / 73 (29.8)	43 (16.4) /36 (13.7)	<.001
C1/C2	75 (30.6) / 4(1.6)	64 (24.4) / 6 (2.3)	.199
Vascular lesions, 1/2	90 (36.7) / 101 (41.2)	84 (32.1) /99 (37.8)	.043
ACEI/ARB, *n*(%)	185 (75.5)	209 (79.8)	.286
Glucocorticoids/immunosuppressors, *n*(%)	134 (54.7)	126 (48.1)	.081
Follow-up duration, months	25.0 (18.0, 32.0)	26.0 (21.0, 35.0)	.044
ESKD, *n*(%)	39 (15.9)	22 (8.4)	–
Endpoint event, *n*(%)	49 (20.0)	27 (10.3)	–

BMI: Body Mass Index ; EGFR: estimated glomerular filtration rate; Units: 15 mL/min/1.73 m^2^; M: mesangial hypercellularity; E: endocapillary hypercellularity; S: segmental glomerulosclerosis; T: tubular atrophy/interstitial fifibrosis; C: cell/cell fiber crescentic lesions; Vascular lesions 1: arteriolar wall thickening/lumen narrowing; 2: merge glassy changes; ACEI/ARB: Angiotensin-Converting Enzyme Inhibitors/Angiotensin Receptor Blockers; ESKD: end-stage kidney disease; Endpoint event: a 50% decrease in eGFR or ESKD; The categorical variable data was expressed as a rate (%). Student’s *t*-test or the chi-square test was used to compare the values of the two groups.

The median follow-up period was 25.0 months for patients with ischemic renal injury. The end-point event was defined as a 50% reduction in eGFR or development of ESKD. The end-point event occurred in 49 (20.0%) patients with ischemic renal injury, comprising 39 ESKD cases (15.9%). In comparison, the median follow-up was 26.0 months for patients without ischemic renal injury. The end-point event occurred in 27 (10.3%) patients without ischemic renal injury, comprising 22 ESKD cases (8.4%) (Table 5). Kaplan-Meier survival analysis revealed that patients with ischemic renal injury exhibited lower cumulative renal survival (Log-rank χ^2^ = 11.352, *p* = .001; Figure 2(C)). Adjusting for gender, age, proteinuria, renal function, MESTC pathology, and treatment, hypertensive IgAN patients with ischemic renal injury exhibited an increased risk of a renal end-point event compared with hypertensive IgAN patients without ischemic kidney injury (HR = 1.889; 95% CI: 1.124–3.178; *p* = .016; Table 6).

**Table 6.** The association between ischemic renal injury and poor prognosis in IgA nephropathy patients with hypertension.

Model	HR	95%CI	*p* value
Model 1	2.138	1.336–3.422	.002
Model 2	1.717	1.068–2.758	.026
Model 3	1.952	1.167–3.264	.011
Model 4	1.889	1.124–3.178	.016

Model 1: Univariate analysis showed that the age and sex of IgA nephropathy patients with ischemic renal injury and non-ischemic renal injury were matched; Model 2: eGFR and 24 h urinary protein were corrected on the basis of model 1; Model 3: mesangial and endocapillary hypercellularity, segmental glomerulosclerosis, and tubular atrophy/interstitial fifibrosis, and cell/cellular fibrous crescents were corrected on the basis of model 2; Model 4: the treatment of Angiotensin-Converting Enzyme Inhibitors/Angiotensin Receptor Blockers or glucocorticoids/immunosuppressors were corrected on the basis of model 3.

## Discussion

Ischemic renal injury is characterized by a complex cascade of events involving small-arterial lesions affecting the glomerular capillary network, followed by glomerular ischemic shrinking, sclerosis, and progression to global glomerulosclerosis and tubular atrophy, resulting in impaired renal function [10]. In China, IgA nephropathy represents the most common form of primary glomerulonephritis, while hypertension remains the most prevalent clinical manifestation in patients with IgAN [11–12]. Increasing numbers of studies suggest that there is an increased prevalence of hypertension in patients with ischemic renal injury [5].

Although ischemic renal injury is common in IgAN patients with hypertension, increasing numbers of normotensive IgAN patients are also increasingly presenting with ischemic renal injury. There are few previous studies of the clinical and pathological characteristics of such patients. Most importantly, there is some debate about whether active intervention is required in these patients and whether ischemic renal injury affects the long-term prognosis of normotensive IgAN patients. The present study investigated the clinicopathological characteristics and prognosis of IgAN patients with ischemic renal injury, with or without hypertension. We analyzed the clinicopathological characteristics and prognostic indicators of all IgAN patients with ischemic renal injury and found that IgAN patients with ischemic renal injury had a higher proportion of hypertension, higher creatinine, uric acid, and a higher proportion of ischemic glomerulosclerosis, renal tubular atrophy/interstitial fibrosis and vascular lesions than those without ischemic renal injury. Increasing evidence indicates that the kidneys receive approximately 20% of cardiac output to maintain glomerular filtration and excrete waste, but consumption of only a small fraction of the oxygen, increasing their susceptibility to ischemic injury due to hypoxia [13]. However, the etiology and mechanism of ischemic renal injury remain elusive. In addition to hypertension, a number of studies have suggested that obesity can cause alterations in renal hemodynamics, thereby, causing obesity-related renal injury and leading to renal ischemia [14]. Hyperuricemia can induce microvascular damage and cause microvascular renal ischemia and associated tissue damage [15]. In addition, excessive complement activation is also believed to be involved in the occurrence of IgAN microvascular lesions and progression to renal ischemia [16]. Once renal ischemia occurs, insufficient renal blood perfusion on one hand can cause impaired glomerular filtration, while on the other hand, reduced capillary blood flow around the renal tubules can lead to renal tubular atrophy/interstitial fibrosis [17]. Therefore, patients with ischemic renal injury display inferior renal function, more severe ischemic glomerulosclerosis, tubular interstitial injury and vascular lesions than patients without ischemic renal injury. Notably, we also found that patients with ischemic renal injury exhibited lower cumulative survival. In addition, ischemic renal injury was found to be a risk factor for renal prognosis in all IgAN patients. Previous studies have indicated that renal tubular atrophy/interstitial fibrosis is a risk factor for the progression of IgAN to ESKD [18–19]. In this study, a significantly higher proportion of IgAN patients with ischemic renal injury had renal tubular atrophy/interstitial fibrosis, and their pathological findings revealed a serious reversal of ischemic lesions and inferior prognosis. This also suggests that in clinical practice, where IgAN patients have combined ischemic renal injury, there may be need to be concerned about the influence of ischemic renal injury on the long-term prognosis of the patient.

Consistent with previous studies that have indicated that not all IgAN patients with renal vascular disease have hypertension [20], the results of the present study also suggest that IgAN patients with normal blood pressure account for 28.8% of those with ischemic renal injury. We used subgroup analysis for hypertensive and normotensive IgAN patients with ischemic renal injury. However, no significant difference in cumulative survival between the two groups of normotensive IgAN patients was observed in the present study. We also found that ischemic renal injury was not a risk factor for end-point events in normotensive IgAN patients. Furthermore, we also analyzed the prognosis of hypertensive IgAN patients with ischemic renal injury. We found that hypertensive IgAN patients with ischemic renal injury exhibited lower cumulative survival. In addition, ischemic renal injury was found to be a risk factor for renal prognosis in hypertensive IgAN patients. Although renal tubular atrophy/interstitial fibrosis is a risk factor for the progression to ESKD in IgAN patients [18–19], the proportion and the severity of ischemic glomerulosclerosis, renal tubular interstitial lesions and vascular lesions in normotensive IgAN patients was lower than that in hypertensive patients. We hypothesize that the lighter ischemic lesions were likely to be reversed following treatment. Thus, the long-term prognosis of normotensive IgAN patients remains unaffected. Of note, the follow-up period was short, and normotensive IgAN with ischemic renal injury is a chronic progressive disease. Therefore, additional studies with extended follow-up periods are warranted to accurately evaluate the long-term prognosis of such patients. In addition, studies have shown that long-term hypertension can directly cause glomerular arteriole damage in which the RAS system becomes activated and inflammatory mediators are released. Interstitial inflammation and microcirculatory disorders develop, inducing hypoxia and renal ischemia [21–22]. Of note, the prevalence of hypertension in patients with primary IgAN is 63.3% [23]. In the present study, we found that IgAN patients with ischemic renal injury had a higher prevalence of hypertension (71.2%). Therefore, when ischemic renal injury occurs in hypertensive IgAN patients, hypertension in concert with other factors can cause renal ischemia, leading to worse renal filtration, more severe renal tubular interstitial injury and poor prognosis. Clearly, Previous studies have indicated that hypertension is a risk factor for the progression of IgAN to ESKD [24–25]. Long-term poor blood pressure control causes hemodynamic changes in the renal microcirculation, which may aggravate a renal injury and lead to poor renal prognosis, while good blood pressure control can delay progression in patients with CKD [26]. This also suggests that clinicians should strengthen the management of hypertensive IgAN patients with ischemic renal injury by actively controlling blood pressure to delay the progression of renal disease.

In conclusion, ischemic renal injury can also be associated with normotensive IgAN. Ischemic renal injury may not affect the prognosis of normotensive IgAN patients, but the prognosis remains poor for hypertensive IgAN patients. Therefore, increased attention should be paid to the management of hypertensive IgAN patients with ischemic renal injury to actively control blood pressure, improve ischemia, and delay the progression of renal disease.

## Study limitations

The study had a number of limitations that should be acknowledged. Firstly, the study was a single-center retrospective study, and thus results may not be generalized to other ethnic groups due to geographical variability in clinical outcomes. Secondly, the follow-up time was short, and IgAN with ischemic renal injury is a chronic progressive disease. Thus, additional studies with extended follow-up periods are warranted to more accurately evaluate the long-term prognosis of IgAN patients with ischemic renal injury.
